# Wearable Bluetooth Triage Healthcare Monitoring System

**DOI:** 10.3390/s21227586

**Published:** 2021-11-15

**Authors:** Caitlin Polley, Titus Jayarathna, Upul Gunawardana, Ganesh Naik, Tara Hamilton, Emilio Andreozzi, Paolo Bifulco, Daniele Esposito, Jessica Centracchio, Gaetano Gargiulo

**Affiliations:** 1 School of Engineering, Design and Built Environment, Western Sydney University, Kingwoods, NSW 2751, Australia; U.Gunawardana@westernsydney.edu.au; 2 MARCS Institute for Brain, Behaviour and Development, Western Sydney University, Milperra, NSW 2560, Australia; t.jayarathna@westernsydney.edu.au; 3 College of Medicine and Public Health, Flinders University, Bedford Park, SA 5042, Australia; ganesh.naik@flinders.edu.au; 4 School of Electrical and Data Engineering, University of Technology Sydney, Ultimo, NSW 2007, Australia; Tara.Hamilton@uts.edu.au; 5 Department of Electrical Engineering and Information Technology, University of Naples Federico II, 80138 Naples, Italy; emilio.andreozzi@unina.it (E.A.); paolo.bifulco@unina.it (P.B.); daniele.esposito@unina.it (D.E.); jessica.centracchio@unina.it (J.C.); 6 Ingham Institute of Applied Medical Research, University of New South Wales, Liverpool, NSW 2052, Australia

**Keywords:** triage, tele-triage, electronic stethoscope, piezoelectric, cardiac, respiratory, wireless

## Abstract

Triage is the first interaction between a patient and a nurse/paramedic. This assessment, usually performed at Emergency departments, is a highly dynamic process and there are international grading systems that according to the patient condition initiate the patient journey. Triage requires an initial rapid assessment followed by routine checks of the patients’ vitals, including respiratory rate, temperature, and pulse rate. Ideally, these checks should be performed continuously and remotely to reduce the workload on triage nurses; optimizing tools and monitoring systems can be introduced and include a wearable patient monitoring system that is not at the expense of the patient’s comfort and can be remotely monitored through wireless connectivity. In this study, we assessed the suitability of a small ceramic piezoelectric disk submerged in a skin-safe silicone dome that enhances contact with skin, to detect wirelessly both respiration and cardiac events at several positions on the human body. For the purposes of this evaluation, we fitted the sensor with a respiratory belt as well as a single lead ECG, all acquired simultaneously. To complete Triage parameter collection, we also included a medical-grade contact thermometer. Performances of cardiac and respiratory events detection were assessed. The instantaneous heart and respiratory rates provided by the proposed sensor, the ECG and the respiratory belt were compared via statistical analyses. In all considered sensor positions, very high performances were achieved for the detection of both cardiac and respiratory events, except for the wrist, which provided lower performances for respiratory rates. These promising yet preliminary results suggest the proposed wireless sensor could be used as a wearable, hands-free monitoring device for triage assessment within emergency departments. Further tests are foreseen to assess sensor performances in real operating environments.

## 1. Introduction

Triage nurses assess patients at the emergency department (ED); their role is primarily to determine the severity of each patient case. The word triage is derived from the French word “trier” as a battlefield sorting process implemented in 1792 by the surgeon in chief to Napoleon’s Imperial Guard, Baron Dominique Jean Larrey [1]. Originally employed for sorting of mass casualties, this novel approach of evacuating the injured from the battlefield evolved throughout the conflicts of the 19th and 20th centuries into a sophisticated sorting process for immediate, urgent, and non-urgent cases. The assessment aligns with a sorting process and is based on three main patient conditions as the name indicates. These three main parameters are patient temperature, respiratory function, and cardiac function. Respiratory and cardiac functions are typically assessed together through auscultation of the body using traditional stethoscopes and a manual count estimating the length of respiratory cycle the heart rate in beats per minute. During the auscultation, a thermometer is fitted, body temperature is particularly important for pediatric and geriatric patients.

Triage outcome is based on a standardized scale to differentiate non-life-threatening cases from critical cases. Assessment scales, such as The Australasian Triage Scale (ATS), present a five-point reference system to prioritise waiting time to treatment of each patient ranging from 0 (immediate) to 120 min [2]. Modern triage typically has three stages, including prehospital triage where emergency vehicles are dispatched and prehospital resources are prepared; on site triage by the responding clinician; and thirdly, triage on ED arrival [1]. Assessment should require less than 5 min to be conducted, so as not to delay other patients waiting. The high-pressure nature of the environment means most triage nurses are to conduct these rapid assessments considering clinical history, psychological assessment and discriminate between category urgencies making it inherently complex. In addition to the initial assessment, triage information must be continually updated to reflect the evolving patient’s condition and consequently update the ongoing responses. According to recent studies, presentation to ED is increasing globally, as example limited to Australia this number is currently estimated in over 22,000 patients per day [3]. Due to increased financial limitations of the health system, resources are being stripped down [1] leading to an increase demand on staff and, thus, led to the integration of telemedicine and wireless tools into the triage assessment [1,4]. By definition Telemedicine is the employment of telecommunications and information technology for remote medical practices that include diagnosis, patient monitoring and data sharing to facilitate the collaboration in diagnosis and treatment of disease [5]. Wearable monitoring systems play a key role within modern telemedicine approaches and have received significant attention in the implementation of modern triage assessment systems. Inclusion of these types of remote monitoring systems assist with standardizing and strengthening coordinated multidisciplinary medical approaches, particularly in mass casualty situations [6].

Established international triage systems based on scales, such as The Manchester Triage Scale (MTS), introduced in 1997, aims to reduce the subjectivity in assessment via the implementation of broader scale algorithms [4]; or the similar 5-level triage algorithm “Emergency Severity Index” (ESI) introduced into the American ED’s in the late 1990’s [7]; have been linked to poor sensitivity and specificity [1]. By combining these well-established multileveled scales with integrated hardware technology and implemented within ED’s could greatly assist the flow and continued/continuous monitoring of patients in care; in other words, a wearable and wireless approach to triage assessment can enhance patient information collection, analysis and interpretation by improving information presentation and facilitating decision making processes [5].

Indispensable tool for triage is a stethoscope. Auscultation of bodily sounds makes easy to assess cardiac and respiratory rhythms; since their inception in 1816, stethoscopes have been an essential tool in medical assessments to perform mediate auscultation [8]. Stethoscopes are typically used as the first line of checking respiratory and cardiac functioning before other monitoring methods such as application of Electrocardiogram (ECG) electrodes which requiring intimate skin contact at precise locations results burdensome to the patient. ECG reflects the electrical properties of the heart however it is not uncommon for structural abnormalities and defects to present themselves as abnormal sounds and murmurs [9]. Graphical record of the heart sounds waveforms called Phonocardiograms (PCG) can be used to assess changes in heart function.

The original stethoscope concept consisted of a brass chest piece and hollow wooden tube has slowly evolved into two main designs being the Y-tube having tubing that splits to each earpiece and Sprague-Rappaport design which has two independent tubes to each earpiece [10]. The chest piece of traditional stethoscopes also improved in design over the past century with two sides used to transmit different sound wavelengths; the diaphragm is used to transmit higher frequencies, while the bell side to transmit lower frequencies [11].

Traditional stethoscopes will attenuate and transmit mechanical distortions picked up from the chest piece using the standing wave phenomenon and some mechanical filtering is conducted along the tubing [12]. Mechanical filtering is provided by 25–30 cm length tubing, which can be constructed with neoprene, plastic or even latex, meaning there can be significant variation in frequency response between construction types [10,13]. Moreover, tubing can add additional resonance to the already present diaphragm resonance (~900 Hz) [13], meaning that resonant peaks do not represent simple harmonics. A study by Ertel et al. [14] reports that uneven frequency response with resonant peaks can lead to corruption of the acoustical signal noting that even a 3 dB attenuation can result in significant information loss.

Interpretation of the sound depends entirely upon the listeners’ perception/experience and can be heavily influenced by the surrounds as well as the acoustical properties of the listener’s ear, Resulting into a compounded signal distortion. It is well-known that bodily sounds and murmurs have low acoustical spectral energy, age-related reduction in auditory sensitivity has been linked to misinterpretation due to undetected frequencies [13]. The most sensitive range for human hearing is typically ranges from 1000~3000 Hz which can be particularly problematic for identifying cardiac sounds as clinically valuable sounds lie between 20~60 Hz [15]. These lower frequencies hold crucial diagnostic information and can be easily overlooked or missed during the assessment.

Antiquated designs are also subject to poor sealing and shock resistance in the tubing connection, making them particularly susceptible to signal corruption from environmental noise and thus making them far less reliable in triage assessment [16]. Other noise artefacts within the signal can be generated from poor contact between the patient and chest piece and loose-fitting earpieces causing leaks and reducing perception of cardiac or respiratory events [17]. As a response, Electronic Stethoscopes (ES) have emerged in the past few decades as a viable solution to the limitations of the traditional designs. Condenser microphones and piezoelectric elements, coupled with a power amplifier to control audio quality and bandwidth, are typically used to replace the bell-diaphragm components. Having higher acoustical isolation, they are ideal for use in highly noisy environments. However, their high cost which can range from several hundred to several thousand AUD make them less suitable for use in ED, where contamination and damage can easily occur [18]. A study by Pinto, et al. [19] also concluded that whilst features may vary between leading market ES, their performance were similar, showing advanced features are not essential for effective use in triage assessment. A low cost specifically triage assessment design, is, therefore, more desirable than the implementation of costly electronic stethoscopes marketed towards specialist practitioners.

As triage assessment is a means to determine the urgency of cases rather than the complexity of the patient’s condition, new and emerging technologies can assist in the growth of patient information recording and sharing. Consequentially, there has been a push for an engineering solution that can assist and enhance the efficiency in triage assessments to ensure timely medical intervention. Implementation of a small-scale electronic stethoscope, tailored to operate wirelessly via the central triage desk, could be used to efficiently monitor multiple patients within the ED. Integration of PC or mobile connectivity via wireless technology can further facilitate the telemonitoring of multiple physiological vital signs through simple, low-cost sensor design. Wearable systems are more sought after for implementation in these situations, where a modular device can continuously show and record patient cardiac and respiratory function in real time. Real time analysis can serve as an advanced method for the identification of key events and the removal of extraneous information, both of which are essential in effective triage assessment. Such analysis can include analysis within the frequency domain over the typical time domain as the respiratory rate is typically around 12–20 breaths per minute or 0.1–0.3 Hz [20], while typical heart rate is 60–100 beats per minute (BPM) or 1–1.67 Hz [21]. Frequency domain analysis, such as the implementation of the Fast Fourier Transform (FFT), can quickly and easily depict respiratory and cardiac function by extracting the main characteristic information that would typically be embedded in time series structure [22]. Cardiac analysis can be performed in both time and frequency domains, for the analysis of the R-to-R interval of the QRS complex of the electrocardiogram (ECG) which can then be used to describe any physiological changes of a patient. The significance of detected R wave in ECG and its corresponding mechanical signal from contraction of the heart muscle, play an important role in the monitoring and diagnosis of patients admitted to the ED. Further improvements of phonocardiography representation through implementation of FFT analysis have been shown by Sumarna et al. [23].

By enabling wearable hand free design, typically time-poor triage nurses and paramedics can continue to perform triage assessment efficiently and may achieve quicker response time to any change in physiological conditions of the patient. Additionally, wearable and personal technologies allow patients to be allocated their own devices during hospital admission meaning more objective assessments can be conducted inclusive of previous patient data. Improving the availability and sharing of quantitative data ensures that comprehensive assessment for correct treatment and diagnosis can be provided [24,25,26].

This paper presents the design of a small-scale tele-triage sensor to monitor respiratory and heart function through the implementation of wireless technology. Small-scale wearable devices that reduce patient discomfort are at the forefront of triage care. Additionally, a push for solutions that can provide remote real-time information from patients is desired to remove the laborious mediate form of auscultation. Patient recordings shown within this paper have been taken from three locations on the body being the suprasternal notch, the radial pulse of the wrist and the apex of the heart. With each of these recordings, both ECG and respiration were also acquired to assess the quality of the signal detected from the proposed sensor.

## 2. Materials and Methods

We propose a wearable, wireless (Bluetooth 5) electronic stethoscope inspired triage system designed around a small piezoelectric sensor completed by a medical grade electronic thermometer, an integrated 3-axis accelerometer to capture body potion and posture and an optional ECG lead.

### 2.1. Design of the Bluetooth Device

#### 2.1.1. Piezoelectric Sensor and Skin Interface

**Figure 1:**
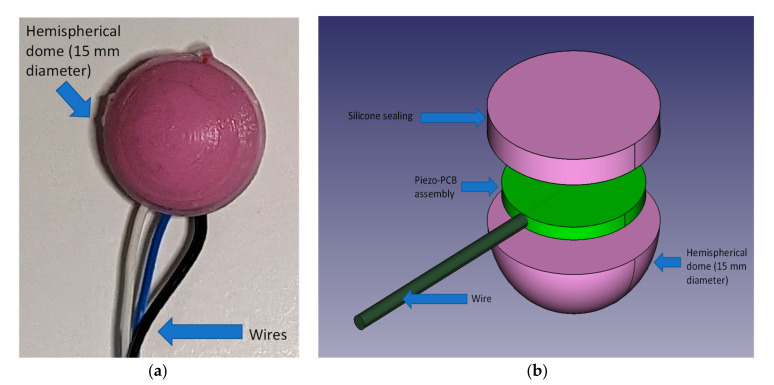
Domed Silicone Enclosure; (**a**): picture of the actual assembly; (**b**): assembly exploded view (not to scale).

At the core of our sensor there is a rigid 12 mm diameter piezoelectric element [27] coated in quickset Pinkysil^®^ (sourced from BARNES Australia) molding silicone to ensure complete coverage and support. The use of a dome-shaped mechanical coupler demonstrated better performance, ensuring a superior and more stable mechanical coupling [28,29]. The Pinkysil^®^ silicone was selected due to its suitability for direct skin contact and rapid set time of 15 min [30]. In a previous study, the Pinkysil^®^ was successfully tested and preferred to other materials for its superior acoustical performances, skin compatibility as well as its mechanical compliance and waterproofing properties [31]. The necessary electronics amplifier is housed on a 12 mm diameter circular printed circuit board (PCB). The inclusion of this PCB, aside providing the necessary sensor with the conditioning circuit, also add structural support to the sensor assembly. The complete assembly weighs less than 10 g and can be held against the skin via an adjustable elastic strap or by a piece of medical tape, both solutions make the sensor wearable and provide uniform pressure to the active area to ensure mechanical vibrations are adequately transmitted to the sensing element. Additionally, the hemispherical shape and homogenous silicone material used for the dome to create tissue subsidence with low irritation and reduced patient discomfort. Figure 1 depicts the sensor assembly; in Figure 1a we present a photograph of one of the prototypes, in Figure 1b the exploded view of the sensor assembly. Manufacture of the sensor is relatively easy, we have manufactured (3D printing) a mold composed by a hemisphere of 15 mm diameter sunk 3 mm with a channel to enable both passage of the wire and silicone excess flow. The manufacture requires four steps:

(1) Pre-assembly and test of the piezo disk on the support PCB
(2) Pre-pouring of silicone in the hemispherical part of the mold
(3) When silicone starts to set (approximately ½ of the curing time) the assembly prepared at step 1 is firmly pressed is the still soft silicone having care placing it in the center and to run the wire in the dedicated channel
(4) Pour additional silicone to cover and seal the sensor assembly inside the dome. The sealing is achieved by the silicone sticking onto itself over the edges of the piezo-PCB assembly.

#### 2.1.2. Sensor Signal Conditioning

Piezoelectric sensors are inherently high impedance signal sources; hence they require specific conditioning and appropriate selection of high input impedance amplifiers for implementation. Additionally, they show high resonance peaks within their frequency response from the dynamic loading and, as such, are recommended to be conditioned to operate below their mechanical resonant frequency [32].

The two most common conditioning circuits for piezoelectric elements are either the voltage mode configuration or the charge amplifier configuration. Transimpedance or voltage mode amplifiers are simple current to voltage converter based on low-drift op-amps when low-frequency signals are of interest [32]. The second type is a charge amplifier, whose function is to obtain a voltage proportional to the charge seen by the amplifier input to produce a low output impedance. Hence, contrary to its name it does not amplify the electric charge seen by the input but is a charge-to-voltage converter. This configuration is best used in applications where the sensor is remote to the conditioning circuit.

The configuration selected for this project was designed as a bespoke hybrid between the two and considered the mechanical resonant properties of the piezoelectric element being 9000 ± 500 Hz specified by the manufacturer datasheet [27]. In series with the sensor was a capacitor with a parallel resistor shunted to the ground, which provided DC blocking in the form of a high-pass filter. These passive components values were calculated using information from the datasheet based on both the capacitance and resistance of the element and were set to 10 nF (C1) and 3.9 MΩ (R3), respectively. Since respiration causes larger and slower displacements and stretches of the chest wall as compared to the small oscillations induced by the heart beats [33], it results in low-frequency electrical signals with much higher amplitudes than those resulting from cardiac activity. Thus, the spectral power of the overall open circuit voltage produced by the piezoelectric sensor is characterized by high values at low frequencies and rapidly decreases towards higher frequencies. The acquisition of such a signal would require a very high dynamic range, which could not be accomplished by the 12-bit analog-to-digital converter involved in this study. Therefore, an 8 Hz cut-off frequency was set to provide proper amplification of the small cardiac-related components and, at the same time, to prevent amplifier saturation due to the large respiration-related components. This is then fed directly into the non-inverting input of the amplifier. A gain setting resistor was then used to amplify the signal and provide stability to the circuit. This design ensured both stability, independent gain setting function and optimization by eliminating the potential mechanical resonance early on within the signal path. This configuration also reduced the necessity for the sensor to be placed close to the conditioning circuit. Amplification at the first stage is achieved by tailoring the resistor R4 to 330 kΩ.

**Figure 2:**
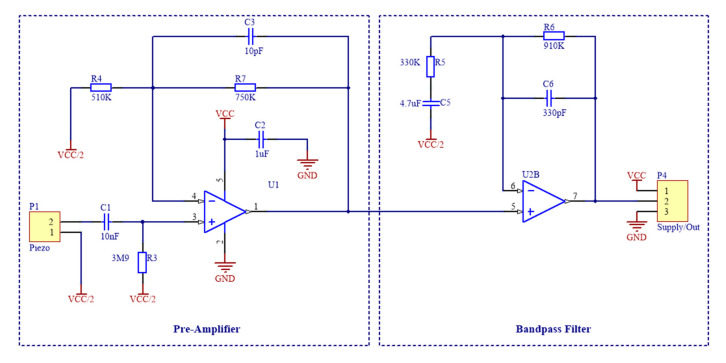
Schematic of the signal conditioning circuit, which is composed by a pre-amplifier for the piezoelectric sensor and a cascaded band-pass filter.

As low frequencies are of interest for biopotential analysis, a suitable second stage analogue filter was designed at the signal output from the pre-amplifier as shown in of Figure 2. A simple yet effective first order, non-inverting bandpass filter was selected to perform this operation with a lower cut off frequency of 0.1 Hz and an upper cut off frequency of 500 Hz. This filter also acted as a secondary gain stage to improve dynamic range of the signal.

**Figure 3:**
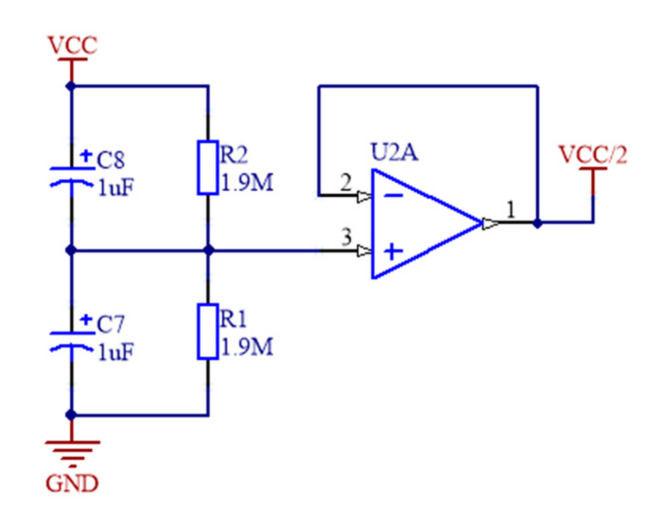
Virtual Ground for power supply splitting from the lithium-ion battery.

As high impedance input from the sensor, a suitable operational amplifier was necessary to perform adequate signal conditioning. The OPA336 series amplifiers were selected for the analogue front end with a single package OPA336 used for the pre-amplifier for the piezoelectric element. One half of a OPA2336 was used to provide the anti-aliasing bandpass filter and the other half to create the virtual ground potential (see Figure 3) that splits in half the voltage supply from a lithium-ion battery for the signal potential reference.

#### 2.1.3. ADC, Body Temperature, ECG and Wireless Connectivity

For a complete wireless sensor design, analogue-to-digital conversion, and wireless connectivity must be incorporated in the design. Our system is designed to be used with a Bluetooth 5 compatible mobile device. The Innocom BM10_AN BLE module with integrated PCB antenna built around the Texas Instruments Simplelink Bluetooth Smart CC2640R2F wireless microcontroller was chosen for the design [34,35]. The optional single ECG lead is provided via the MAX30003 single channel biopotential frontend, the analogue to digital conversion of the acoustic signal is obtained by the Texas Instruments ADS1247 [36,37]; for this application, we sample only one differential channel of the ADS1247, the secondary channel is dedicated for future system expansion, i.e., a secondary acoustic sensor. Both the ECG device and the ADS1247 transmit data at the standardized sample rate of 300 Hz. To complete a full triage device the inclusion of the Maxim integrated MAX30205 human body temperature sensor by was sampled at 6.5 Hz directly by the MCU for Bluetooth streaming to an Android mobile device [38]. The sensor was also cast in the skin safe Pinkysil^®^ silicone to assist patient skin contact under the patient’s armpit or using an adjustable elastic chest strap.

**Figure 4:**
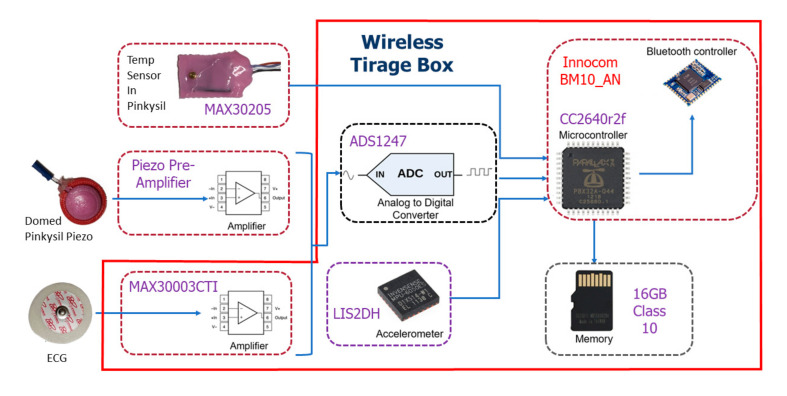
Proposed wireless triage device box with sensor frontends depicting the system architecture of data flow.

A LIS2DH triple-axis accelerometer, which supports up to 16 g acceleration, was sampled at 300 Hz and directly interfaced to the MCU to detect changes in patient body position [39]. This accelerometer system has been used in previous investigations to detect changes in sleep position [40]. All data are recorded to a micro-SD card and could be live-streamed to an Android mobile device using Bluetooth 5 Protocol. Figure 4 depicts the full system architecture of data flow for the proposed device.

**Figure 5:**
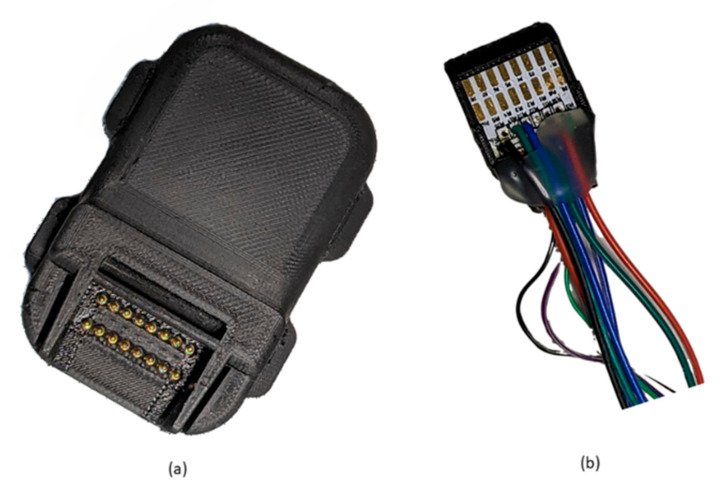
(**a**) enclosed wireless triage box (**b**) wireless triage box connector for the Domed Piezo Sensor, ECG electrodes and Temperature sensor.

The red block encasing some components of the system (see Figure 4) shows the components that are enclosed in a custom made 3D printed enclosure for the wireless triage device box shown in Figure 5a. The piezo sensor with pre-amplifier PCB cast in domed Pinkysil^®^ silicone, ECG electrodes and MAX30005 also in Pinkysil^®^ silicone are interfaced via pogo pins using the connector shown in Figure 5b. The wireless triage box also contains a rechargeable 3.7v lithium-ion battery that can power the full unit for approximately 12 h.

### 2.2. Analysis of Proposed Sensor Performances in Cardiac and Respiratory Monitoring

#### 2.2.1. Data Collection

The proposed sensor was previously compared with an ECG and an electro resistive band (ERB) [40,41,42] to demonstrate its effectiveness in detecting both cardiac and respiratory events, which are necessary to perform triage assessment in EDs. ECG was selected as benchmark for two main reasons, the primary being that it is the healthcare standard for non-invasive measurement of cardiac function and for cardiac monitoring in triage, and the secondary being that it also carries information on respiration that can be used to extract the respiratory rate [43]. Although respiratory rate can be extracted from ECG, the ERB was considered as a more reliable benchmark for respiratory monitoring, as it proved capable of accurately monitor the variations of tidal volume of the lungs during respiration [33,40,41,42].

**Figure 6:**
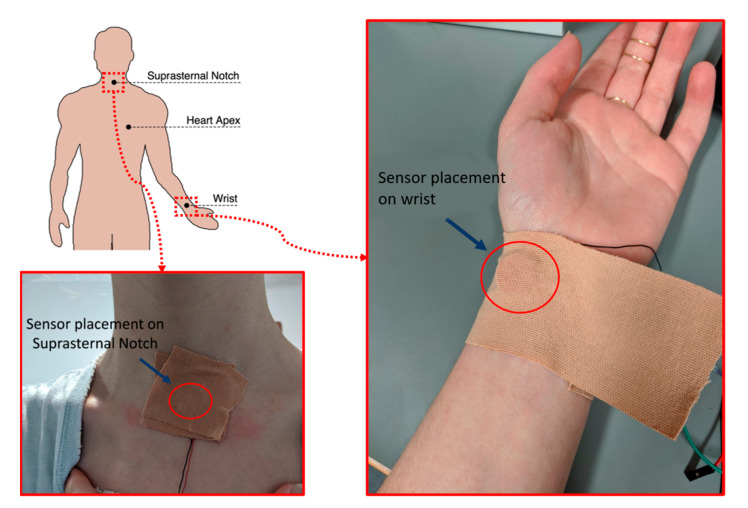
Placement of piezoelectric sensors onto the subject’s body, with details of sensor positioning onto the suprasternal notch and the radial pulse on the wrist.

Simultaneous recordings of the proposed piezoelectric sensor, an ECG and an ERB were acquired on three subjects (co-authors) via a BIOPAC^®^ system (sources from https://www.biopac.com/, accessed on 1 September 2021) [44], composed of an MP160 data acquisition platform, an ECG100C module with disposable Ag-AgCl electrodes, two generic amplifier (no gain) modules AMI100D (for piezoelectric sensors and ERB) and the AcqKnowledge^®^ data acquisition software. All the signals were sampled at 1 kHz and digitized with 16-bit resolution. The ERB was strapped across the volunteer chest as not to obstruct or interfere with the ECG or sensors leads. Three different areas were considered for the acquisition of signals via the proposed sensor, namely the heart apex area onto the chest, the suprasternal notch, and the wrist. The first is undoubtably the location closest to the heart and is also commonly considered for heart sounds auscultation; the second can be used to detect a variety of chronic conditions of the body and is commonly used to take intrathoracic pressure [45]; the third is commonly considered for manual monitoring of heart rate via palpation of the radial pulse and was included in this study also to investigate the feasibility of respiration monitoring from the wrist. Recordings with piezoelectric sensors were taken in pairs with the first sensor placed onto the chest in the heart apex area and the second onto either the suprasternal notch or the wrist. The recorded data were then processed and analyzed in MATLAB^®^. Details of the sensor placement are shown in Figure 6. In Figure 6, our custom sensor is applied with medical tape to ensure the stability of the sensor assembly on the skin in all positions, although the sensor is lightweight and can be held in position on the chest and on the wrist comfortably with an elastic strap, such arrangement resulted uncomfortable for the suprasternal notch. To ensure consistency, for this work, we used medical tape at all positions throughout all the tests.

#### 2.2.2. Pre-Processing

The acquired data were pre-processed before performing heart rate (HR) and respiratory rate (RR) estimation. All raw signals except ECG were processed with a one-pole one-zero high-pass filter (The filter transfer function in polynomial form as it can be entered in MATLAB directly is reported in Appendix A) to remove the DC component [46], this filter yelds a 3 db cutoff frequency of 0.023 Hz. Moreover, the signals recorded onto the chest were also low-pass filtered via of a 4th-order Butterworth filter with a 4 Hz cut-off frequency. The processed sensors signal just obtained were used to extract information on cardiac cycle. Respiration signals were extracted both from piezoelectric sensors and ECG recordings, to compare the respiratory monitoring performances of the proposed sensor also with a pure ECG device. To this aim, the raw signals of the piezoelectric sensors and the ERB were low-pass filtered via a 4th-order Butterworth filter with 0.5 Hz cut-off frequency; the ECG-derived respiration signal (EDR) was extracted from ECG recordings by means of the BioSigKit toolkit [47].

#### 2.2.3. Heart and Respiratory Rates Extraction

Fiducial points for heartbeats and respiratory acts to be used for HR and RR computation were first located in the analyzed signals. In ECG, the R peaks were detected via the well-known Pan and Thompkins algorithm [48], while, in the processed sensors signals for cardiac cycle analysis, the positive peaks following the ECG R peaks were detected via the MATLAB^®^ function “findpeaks”. In all the signals for respiration cycle analysis, the negative peaks were considered as fiducial points and detected via the MATLAB^®^ function “findpeaks”. The detected peaks were analyzed to identify the true positives (TP), the false positives (FP) and the false negatives (FN) with respect to the reference signal (ECG for HR, ERB for RR), which were used to determine the sensitivities and the positive predicted values (PPV) for each signal.

Then, the instantaneous heart and respiratory rates series were extracted from all the signals by excluding the FP and FN peaks. Some recordings were corrupted by motion artefacts, which resulted in very high estimation errors for the corresponding HR values. These values were excluded from the instantaneous HR series.

#### 2.2.4. Statistical Analyses of Heart and Respiratory Rates

The HR series extracted from the piezoelectric sensors signals acquired on the heart apex, suprasternal notch and wrist were compared with the HR series extracted from ECG by means of correlation and Bland-Altman analyses, which were carried out via the MATLAB function “bland-Altman-and-correlation-plot” [49]. The same analyses were performed on the RR series extracted from the sensor’s respiration signals and the EDR, which were compared with the RR series extracted from the ERB signals.

## 3. Results

### 3.1. Peak Detection Method

**Figure 7:**
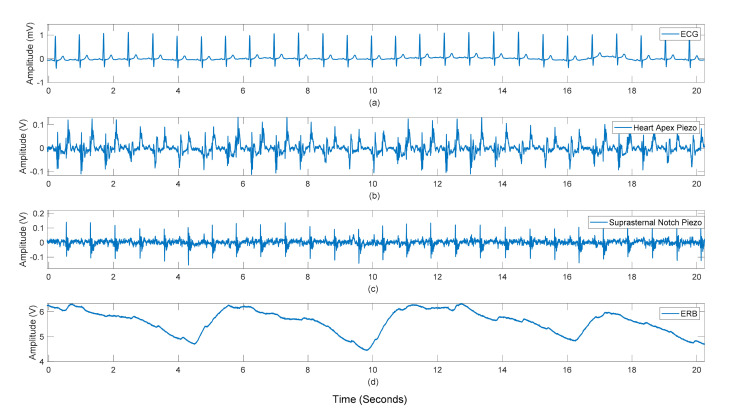
Subject #3 dataset: Raw recordings from (**a**) ECG signal, (**b**) sensor placed on the heart apex (**c**) sensor on the suprasternal notch (**d**) the respiration band around the chest.

**Figure 8:**
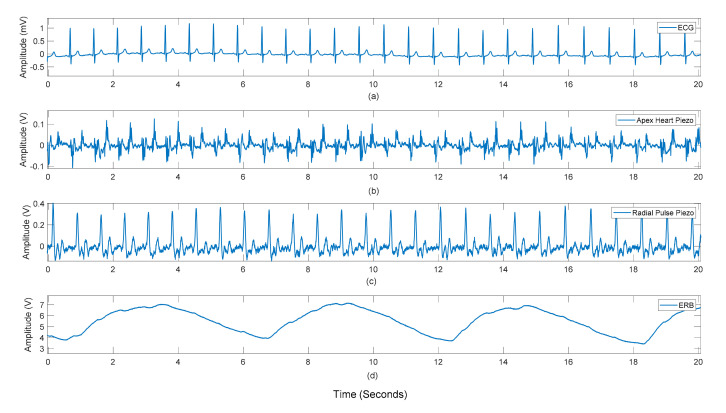
Subject #3 dataset: Raw recordings from (**a**) ECG signal, (**b**) sensor placed on the heart apex (**c**) sensor on the radial pulse of the wrist (**d**) the respiration band around the chest.

Figure 7 and Figure 8 show the raw data acquired from the sensor as seen by the heart apex, the suprasternal notch, and the wrist, with both ground truths being the ECG and the ERB signal.

**Figure 9:**
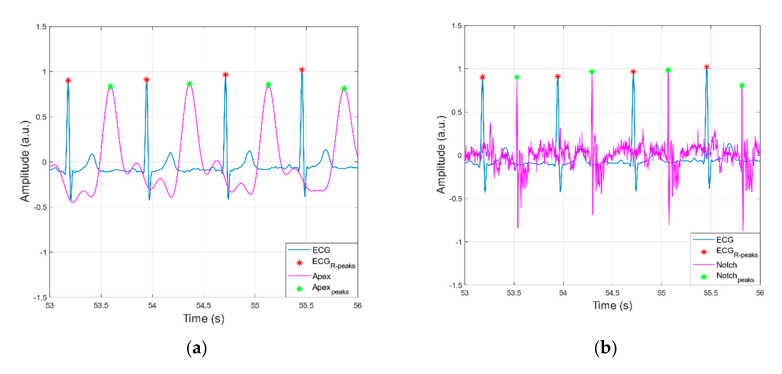

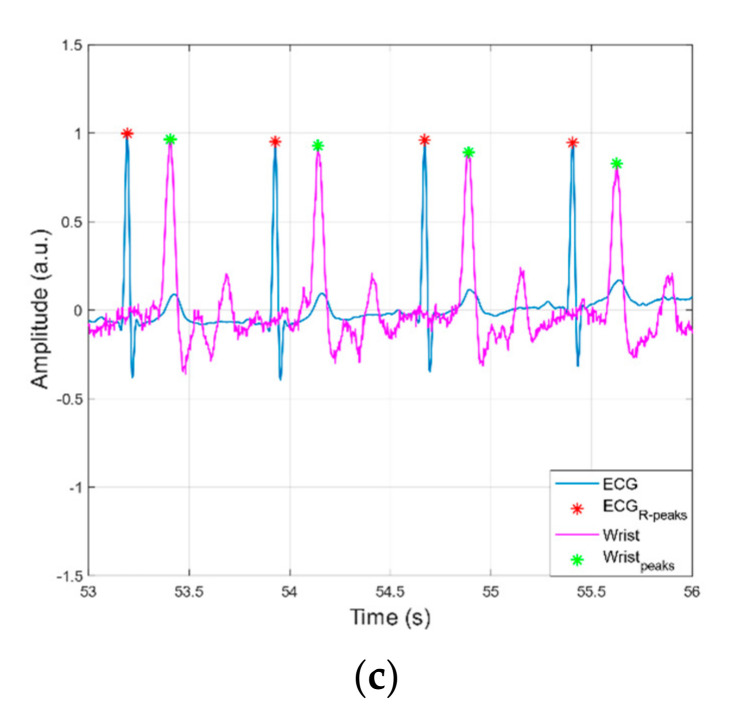
Examples of peaks detection in ECG versus heart monitoring signals acquired via the proposed sensor from: (**a**) heart apex; (**b**) suprasternal notch; (**c**) wrist.

**Figure 10:**
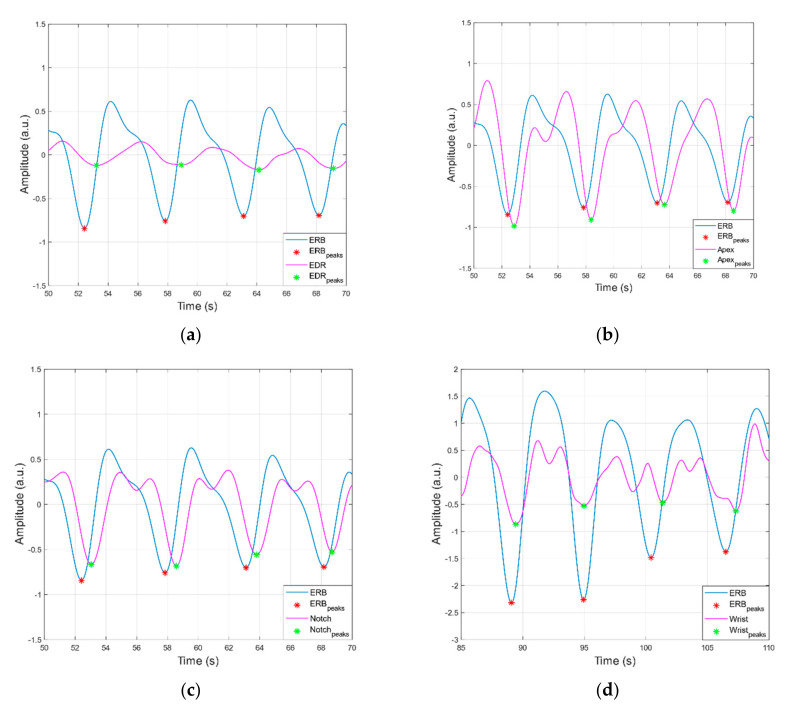
Examples of peaks detection in ERB signal versus: (**a**) EDR and (**b**–**d**) respiration monitoring signals acquired via the proposed sensor from heart apex, suprasternal notch and wrist, respectively.

Figure 9 and Figure 10 report examples of peaks detection in heart and respiration monitoring signals, respectively. In Table 1, statistics on performances of peak detection are reported. In all heart monitoring signals from piezoelectric sensors, 100% sensitivity and almost 100% PPV were achieved, while in respiration monitoring signals, such high performances were attained only in apex and suprasternal notch signals, with substantially lower performances obtained in respiration signals extracted from wrist and ECG (EDR).

### 3.2. Statistical Analyses of Heart and Respiratory Rates

**Figure 11:**
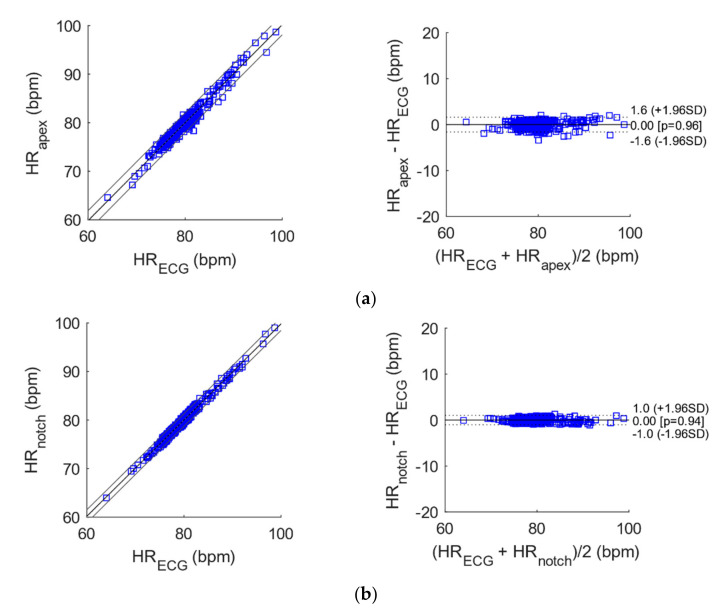

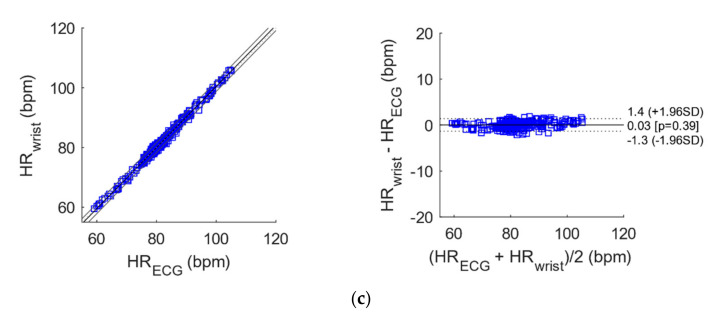
Correlation and Bland-Altman plots for the instantaneous heart rates extracted from ECG versus those extracted from the heart monitoring signals acquired by the proposed sensor from: (**a**) heart apex; (**b**) suprasternal notch; (**c**) wrist.

**Figure 12:**
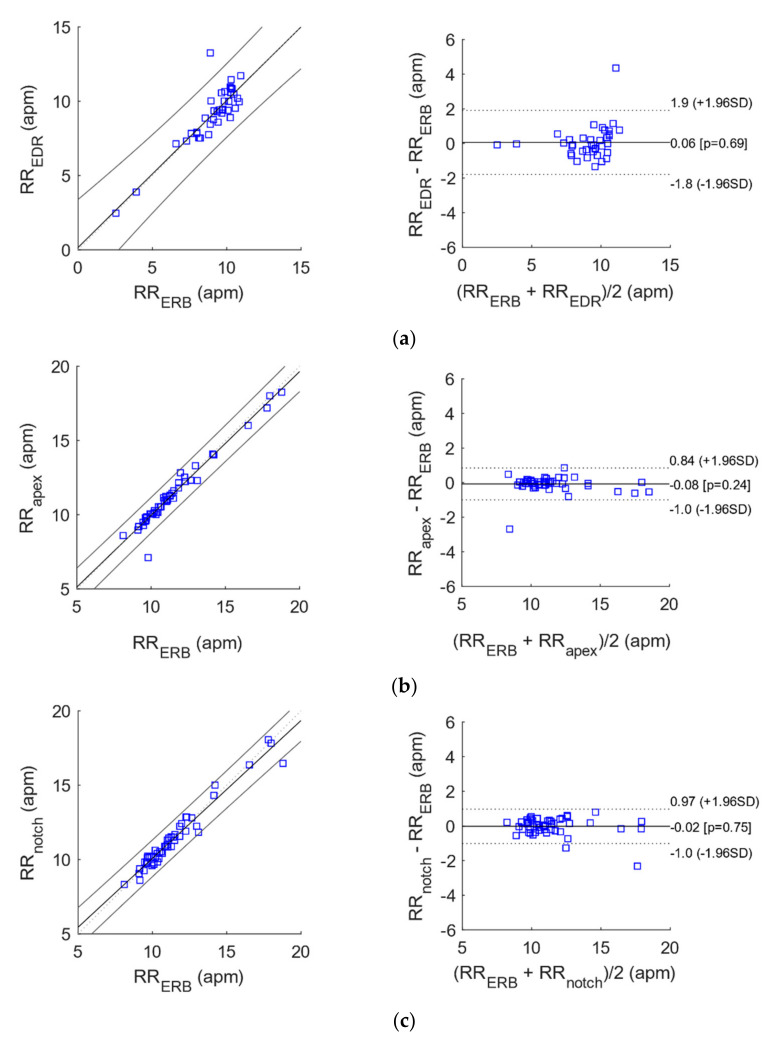

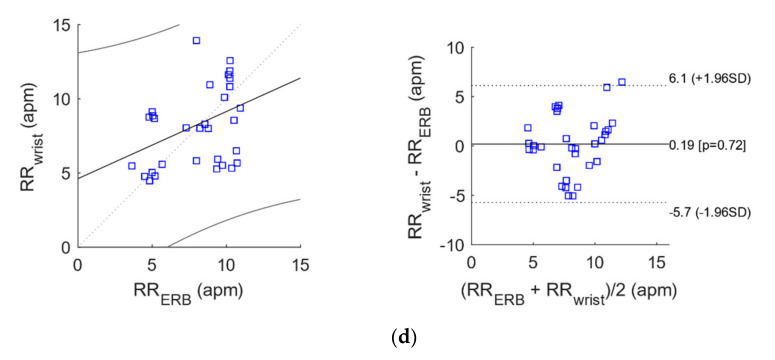
Correlation and Bland-Altman plots for the instantaneous respiratory rates extracted ECG versus those extracted from (**a**) EDR and (**b**–**d**) the heart monitoring signals acquired by the proposed sensor from heart apex, suprasternal notch and wrist, respectively.

The results of correlation and Bland-Altman analyses for heart and respiratory rates estimation are reported in Table 2 and Table 3 and depicted in Figure 11 and Figure 12.

The heart monitoring signals acquired by the proposed sensor from all the three locations provided instantaneous HR series very similar to those provided by ECG, as proved by the almost unit slopes and the very high R^2^ values obtained from correlation analysis. Moreover, they achieved limits of agreement (LoA) of less than 2 bpm, with an almost null bias.

With reference to Table 2 and Table 3, the RR extracted from the apex and suprasternal notch recordings were highly comparable to those provided by the ERB benchmark (R^2^ > 0.95), and achieved better performances with respect to EDR. The RR obtained at the wrist, instead, attained much lower performances.

### 3.3. ADC and Bluetooth Streaming Testing

**Figure 13:**
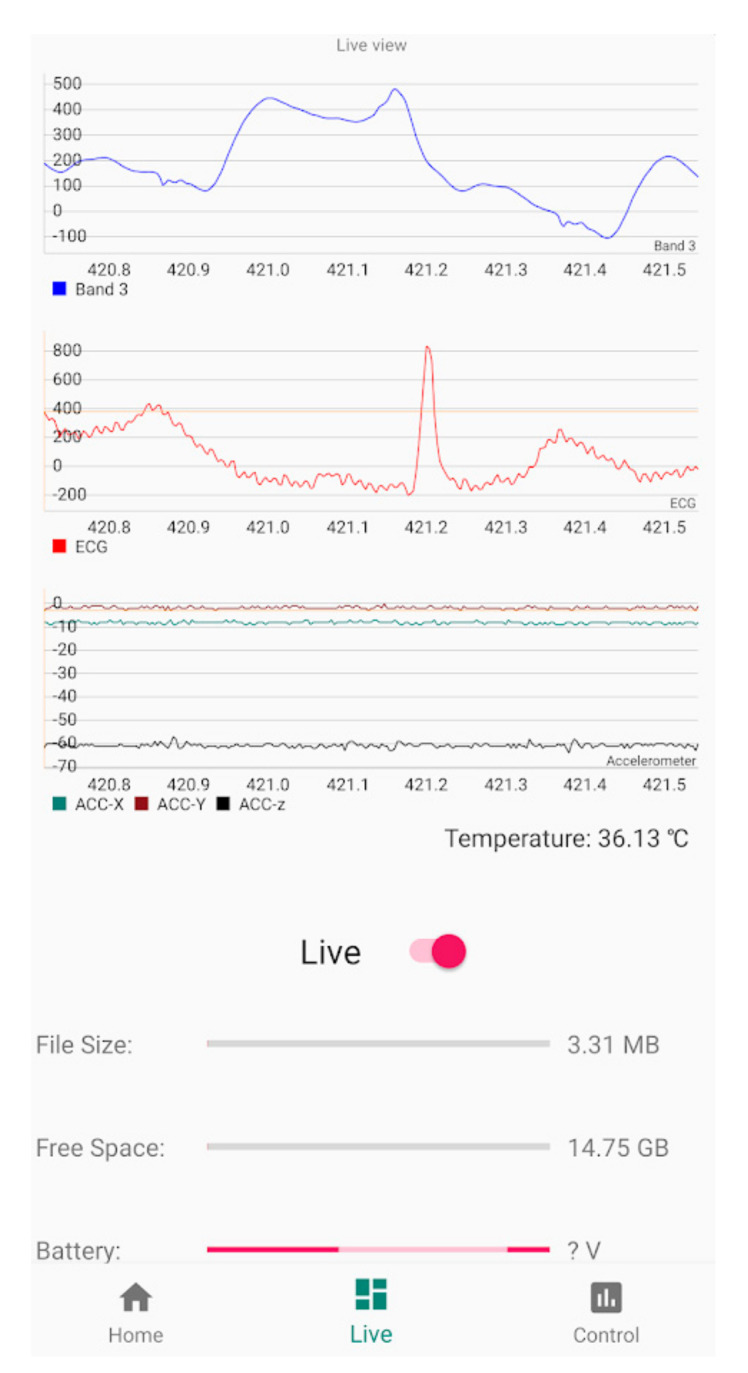
Screenshot (limited to one cardiac beat) taken from live Bluetooth streaming to Android 11 device including the proposed piezo based sensor as Band 3, ECG, X-Y-Z accelerometer data and body temperature from top to bottom. Please note that Y-axis units’ conversion in the prototype App have not been implemented yet.

To verify that the signal was streamed with no loss of data, data were recorded from the patient’s chest and plotted within a mobile app as shown in Figure 13 for approximately one second of live data during one of the stationary tests. Cardiac events corresponding to the R-to-R intervals can be seen as clear heartbeats and the slight baseline wander seen in this window corresponds to the rhythmic respiration. The video recorded by the mobile device during live streaming (approx. two minutes) is available for download, see Supplementary Materials section.

## 4. Discussion

The use of the silicone dome on the sensor was preferred as it improves the mechanical coupling with the subjects’ skin, thus assuring stable and reliable measurements. The semi-rigid silicone enclosure provided sufficient protection for the delicate piezoelectric ceramic element and the PCB which host the analogue frontend, thus making it more robust. The proposed piezo frontend design and reference ECG using the MAX30003 were sent to the ADS1247 ADC before going to the Innocom BM10_AN R2 BLU module for wireless data streaming and storage to a micro-SD card. The MAX30205 body temperature sensor, which was also cast in Pinkysil^®^, and the LIS2DH accelerometer for patient body potion and motion when triaging was sent directly to the MCU module, for future improvement, the positioning of the box should be fixed on the body, e.g., the belt so that the body posture can be drawn from the accelerometer sensor. The ECG, piezo front end, and accelerometer where each sampled at 300 Hz while the temperature sensor was sampled at 6.5 Hz. As such a well-rounded Bluetooth connectivity wireless triage monitoring system has been proposed within this research that would be suitable for implementation in ED and first response events.

The proposed sensor achieved performances comparable to ECG for heart rate measurement in all the three recording areas here considered. The sensor also obtained good performance for respiratory rate measurement with respect to ERB and outperformed the EDR. However, the recordings acquired on the wrist were not reliable for accurate respiratory rate extraction.

These preliminary results suggest that the proposed sensor is viable for patient monitoring, also for triage. It could offer some advantages with respect to conventional ECG and respiratory chest bands:

- it is based on a cheaper sensor and requires a simpler conditioning circuit.
- it requires only one measurement point, which may be positioned onto different body areas.
- it does not need any electrical contact with the body and conductive gel.
- it is much more robust to electromagnetic interferences.
- it needs much simpler processing to extract a respiration signal.

However, these promising results must be confirmed by further studies involving much more healthy subjects and patients, also during real triage assessments in ED’s.

## 5. Conclusions

The sensor proposed in this article has been designed to be used for either short or long-term triage monitoring in EDs. The piezoelectric element was selected as it is an off-the-shelf component that can be easily cast in suitable silicone to improve mechanical coupling. The pre-amplified signal produced was clear and suitable for detection of both cardiac and respiratory events. Although not employed (it was not necessary) for this study, the selected ADC aside having a high bit count over a small voltage range also includes a programmable gain amplifier that we plan to exploit via the App to further amplify the signal where required. Simple processing using common filtering and threshold peak detection allowed to extract instantaneous heart and respiratory rates that were of high sensitivity and comparable to ECG and ERB, respectively. Bluetooth connectivity supports the proposed system to be used as a wearable hands-free monitoring device for triage assessment within emergency departments. Further work on the user interface on the mobile device can be developed, with input from clinicians and practitioners, to create a bespoke interface with essential information displayed in any statistical and or graphical method of choice. Moreover, a comparative study on different dome materials could be accomplished to optimize the impedance matching between the piezoelectric sensor and the skin, with the aim of improving the mechanical energy coupling. Finally, additional analyses are foreseen to compare and/or integrate the proposed sensor with other sensors for cardiac mechanics such as accelerometers adopted in Seismocardiography [50] and Force Sensitive Resistors adopted in Forcecardiography [51].

## Figures and Tables

**Table 1:** Statistics for peak detection in heart and respiration monitoring signals.

	TP	FP	FN	SENSITIVITY (%)	PPV (%)
Apex vs. ECG	374	0	0	100	100
Notch vs. ECG	374	0	0	100	100
Wrist vs. ECG	372	2	0	100	99.5
Apex vs. ERB	51	0	0	100	100
Notch vs. ERB	51	2	0	100	96.2
Wrist vs. ERB	33	12	11	75.0	73.3
EDR vs. ERB	39	1	5	88.6	97.5

**Table 2:** Results of correlation analysis for instantaneous heart and respiratory rates.

	*N*	Slope	Intercept (bpm/apm)	R^2^
Apex vs. ECG	372	1.0098	−0.7775	0.97
Notch vs. ECG	368	0.9906	0.7482	0.98
Wrist vs. ECG	371	1.0221	−1.7724	0.99
Apex vs. ERB	50	0.9673	0.2937	0.96
Notch vs. ERB	50	0.9273	0.8046	0.95
Wrist vs. ERB	32	0.4521	4.6176	0.13
EDR vs. ERB	38	0.9896	0.1565	0.77

**Table 3:** Results of Bland-Altman analysis for instantaneous heart and respiratory rates.

	*N*	Bias (bpm/apm)	Limits of Agreement (bpm/apm)	
Apex vs. ECG	372	0.0021	−1.606472	1.610672
Notch vs. ECG	368	−0.0022	−1.036492	1.032092
Wrist vs. ECG	371	0.0309	−1.331496	1.393296
Apex vs. ERB	50	−0.0781	−0.996752	0.840552
Notch vs. ERB	50	−0.0232	−1.014764	0.968364
Wrist vs. ERB	32	0.1945	−5.728228	6.117228
EDR vs. ERB	38	0.0621	−1.783632	1.907832

## Supplementary Materials

The following are available online at https://www.mdpi.com/article/10.3390/s21227586/s1, Video S1: Mobile device during live streaming.

## Appendix A. DC Removal Filter Matlab Code

b = [1 −1];

a = [1 −0.9996];

% arrays a and b contains the filter coefficients

signal_out = filtfilt(b,a,signal_input);
